# Contribution of *Pseudomonas aeruginosa* Exopolysaccharides Pel and Psl to Wound Infections

**DOI:** 10.3389/fcimb.2022.835754

**Published:** 2022-04-07

**Authors:** Derek Fleming, Brandon Niese, Whitni Redman, Emily Vanderpool, Vernita Gordon, Kendra P. Rumbaugh

**Affiliations:** ^1^ Department of Surgery, Texas Tech University Health Sciences, Lubbock, TX, United States; ^2^ Department of Physics, Center for Nonlinear Dynamics, The University of Texas at Austin, Austin TX, United States; ^3^ Interdisciplinary Life Sciences Graduate Programs, LaMontagne Center for Infectious Disease, The University of Texas at Austin, Austin, TX, United States; ^4^ Burn Center for Research Excellence, Texas Tech University Health Sciences, Lubbock, TX, United States

**Keywords:** biofilm, *Pseudomonas aeruginosa*, wound infection, exopolysaccharides, aggregate, Pel, Psl

## Abstract

Biofilms are the cause of most chronic bacterial infections. Living within the biofilm matrix, which is made of extracellular substances, including polysaccharides, proteins, eDNA, lipids and other molecules, provides microorganisms protection from antimicrobials and the host immune response. Exopolysaccharides are major structural components of bacterial biofilms and are thought to be vital to numerous aspects of biofilm formation and persistence, including adherence to surfaces, coherence with other biofilm-associated cells, mechanical stability, protection against desiccation, binding of enzymes, and nutrient acquisition and storage, as well as protection against antimicrobials, host immune cells and molecules, and environmental stressors. However, the contribution of specific exopolysaccharide types to the pathogenesis of biofilm infection is not well understood. In this study we examined whether the absence of the two main exopolysaccharides produced by the biofilm former *Pseudomonas aeruginosa* would affect wound infection in a mouse model. Using *P. aeruginosa* mutants that do not produce the exopolysaccharides Pel and/or Psl we observed that the severity of wound infections was not grossly affected; both the bacterial load in the wounds and the wound closure rates were unchanged. However, the size and spatial distribution of biofilm aggregates in the wound tissue were significantly different when Pel and Psl were not produced, and the ability of the mutants to survive antibiotic treatment was also impaired. Taken together, our data suggest that while the production of Pel and Psl do not appear to affect *P. aeruginosa* pathogenesis in mouse wound infections, they may have an important implication for bacterial persistence *in vivo.*

## Introduction

Microbes in chronic infections most commonly exist as biofilms; communities of microorganisms dwelling within a matrix made of largely self-produced extracellular substances (EPS), including polysaccharides, proteins, eDNA, lipids and other molecules (Flemming and Wingender, 2010). In fact, biofilms have been estimated to be involved in 80% of all human bacterial infections, and 90% of chronic wound infections (Attinger and Wolcott, 2012; Romling and Balsalobre, 2012). Living within the protection of the EPS matrix is thought to provide microorganisms greatly increased tolerances to antimicrobials and the host immune response (Rogers et al., 2010; Lewis, 2012). These tolerances arise from several mechanisms, including physical and chemical protection by the EPS matrix and reduced metabolic activity of many of the microorganisms in the biofilm. Lowering metabolic activity decreases susceptibility to the majority of antibiotics that target metabolically-active cells (Koo et al., 2017).

Given their prevalence in chronic infection, and the magnitude of their impact on human health, biofilms have been studied extensively over the past several decades. While many great strides have been made in understanding the pathophysiological mechanisms and genetics of biofilm formation and persistence, the vast majority of the work has been performed in artificial, *in vitro* environments with limited clinical relevance. Indeed, it has become clear that biofilm properties vary greatly between *in vitro* and *in vivo* settings, and even across differing infection sites and conditions *in vivo* (Bjarnsholt et al., 2013; Roberts et al., 2015; Ibberson et al., 2017; Redman et al., 2020). It is thus vital to investigate the properties of biofilms *in situ* to better understand their influence on infection. A proper understanding of how and when microbes form biofilms during infection and how these structures are composed is also needed to design new biofilm-targeting therapies.

For many microorganisms, biofilm formation varies considerably both temporally and from environment to environment (Stanley and Lazazzera; 2004, Karatan and Watnick, 2009; Yang et al., 2011; Redman et al., 2020). This is true for *Pseudomonas aeruginosa*, a biofilm-forming opportunistic pathogen that is involved in a wide range of infection types. One key way that *P. aeruginosa* can alter its EPS matrix composition is by differential expression of its three exopolysaccharides, Pel, Psl, and alginate (Ryder et al., 2007; Colvin et al., 2012; Rahman et al., 2021). Exopolysaccharides are major structural components of bacterial biofilms (Flemming and Wingender, 2010) and contribute to numerous aspects of biofilm formation and persistence, including adherence to surfaces, coherence with other biofilm cells, mechanical stability, protection against desiccation, binding of enzymes, nutrient acquisition and storage, and protection against antimicrobials, host immune cells and molecules, and environmental stressors (Flemming and Wingender, 2010). Pel is a cationic polysaccharide, containing acetylgalactosamine and acetylglucosamine sugars, that has been shown to aid in cell-cell adherence, surface attachment, DNA crosslinking, and protection against aminoglycosides (Colvin et al., 2011; Jennings et al., 2015a; Jennings et al., 2015b). Psl is a mannose, glucose, and rhamnose-rich polysaccharide that is also involved in cell-cell interactions and surface attachment (Irie et al; Ma et al., 2007; Byrd et al., 2009). Indeed, it has been suggested that Pel and Psl are structurally redundant, with successful *P. aeruginosa* strains often expressing one or the other, including the common laboratory strains PAO1 and PA14, which produce Psl-dominant and Pel-dominant biofilms respectively (Colvin et al., 2011). Alginate is a mucoid polysaccharide, composed of guluronic and mannuronic acid, that is strongly associated with *P. aeruginosa* lung infection isolates from cystic fibrosis (CF) patients (Franklin et al., 2011). Alginate is thought to aid in biofilm formation and immune evasion, but doesn’t appear to play a significant role outside of the CF lung (Wozniak et al., 2003).

*P. aeruginosa* infection is highly prevalent in chronic wounds, estimated to be present in about 25% of all cases (Wolcott et al., 2016). However, since most *P. aeruginosa* biofilm studies have been performed *in vitro*, little is known about how different exopolysaccharides impact *P. aeruginosa* infection or the host response. In this study, we used a murine chronic wound model, and a selection of *P. aeruginosa* strains with different patterns of exopolysaccharide production, to examine the impact of differential polysaccharide composition on the size and spacing of bacterial aggregates, infection load, antibiotic susceptibility, and interaction with host cells.

Overall, we saw that the lack of Pel and Psl had little effect on the severity of wound infection, as bacterial loads in wounds and wound closure were unaffected. However, we saw significant differences in the spatial properties of *P. aeruginosa* aggregates in wound tissue when Pel and/or Psl were absent. Specifically, the absence of Pel and Psl resulted in much smaller aggregates spaced further apart. We also noted differences in the number of host cells surrounding these aggregates, which could have implications for an immune response. Importantly, the loss of both Pel and Psl affected the ability of *P. aeruginosa* to survive aminoglycoside treatment. These results are important to our understanding of how composition of the biofilm matrix can influence wound infection and bacterial persistence *in vivo*.

## Results

### Neither the Deletion Nor Overproduction of Pel or Psl Affects Wound Infection in a Mouse Model

In theory, the ability to form robust biofilms should confer protection to bacteria from the host immune system during infection. While studies examining *in vivo* biofilm formation and biofilm/host interactions are few, there is some support that the ability to make a biofilm is a fitness attribute and potentially increases virulence *in vivo* (Pestrak et al., 2018). To examine the role of Pel and Psl in wound infections, we used the wild-type (WT) strain PAO1 and 4 mutants derived from PAO1 with deletions in different genes affecting exopolysaccharide production (Δ*pel*, Δ*psl*, Δ*pel*Δ*psl*, Δ*wspF*, **Table 1**).

**Table 1:** Description of the *P. aeruginosa* strains used in this study.

Strain	Mutation	Impact of Mutation on EPS Exopolysaccharide Composition	Reference
PAO1	NA	Wild-type strain originally isolated from wound, capable of producing Psl, Pel and alginate	(Holloway, 1955)
PAO1Δ*pel*	*pelA*; polar mutant of the pel operon	Does not make the polysaccharide Pel	(Kirisits et al., 2005; Borlee et al., 2010)
PAO1Δ*psl*	*pslBCD*; polar mutant of psl operon	Does not make the polysaccharide Psl, overproduces Pel	(Kirisits et al., 2005)
PAO1Δ*pel*Δ*psl*	*pelA* and *pslBCD*; polar mutations	Does not make the polysaccharides Psl and Pel	(Rybtke et al., 2012b)
PAO1Δ*wspF*	In frame deletion of *wspF*	Constitutively over-expresses cyclic-di-GMP, which acts as a signal for biofilm development. Pel, Psl and CdrA overexproduced	(Starkey et al., 2009)

**Figure 1:**
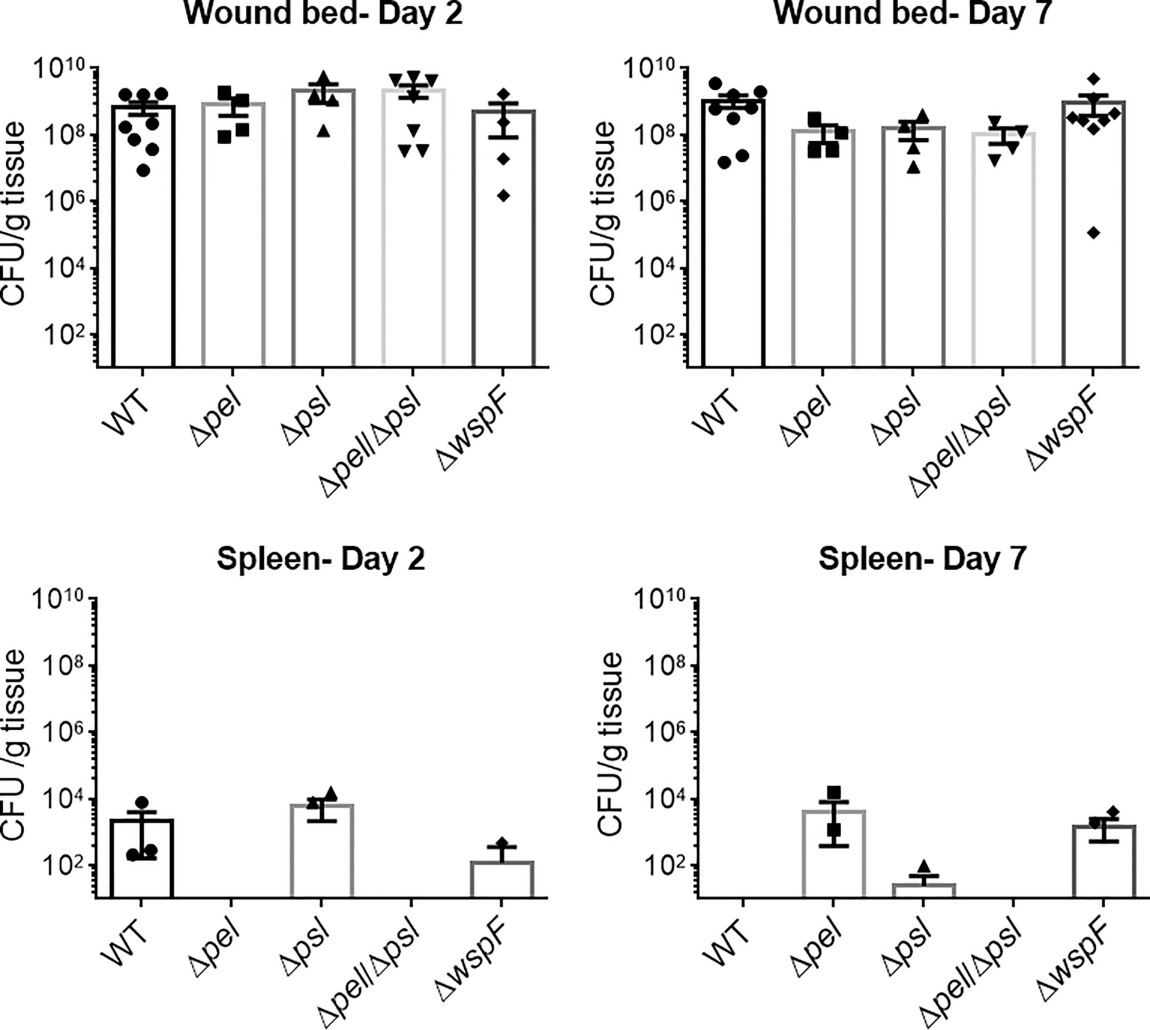
No significant differences in wound or spleen bacterial load were observed between groups. Mice were wounded and infected with indicated *P. aeruginosa* strains. After 2 or 7 days, wound tissue and spleens were harvested and processed for CFU determination. One-way analysis of variance and a Dunn’s or Dunnett’s multiple comparison test was used to test for differences between groups. n=4-8 mice/strain.

The *wspF* gene encodes the regulatory protein of the diguanylate synthase, WspR (Hickman et al., 2005). It has previously been established that deletion of WspF results in the elevated production of the matrix protein CdrA and the pro-biofilm secondary messenger molecule, cyclic-di-GMP, resulting in a constitutive upregulation of Pel and Psl and biofilms with significantly more biomass (Hickman et al., 2005; Rybtke et al., 2012a). Thus we sought to determine if a Δ*wspF* mutant would cause a more virulent or persistent infection *in vivo*. We also sought to determine if the absence of Pel and/or Psl would affect *in vivo* fitness. To test this, mice were administered surgical excision wounds and infected with PAO1 or an exopolysaccharide mutant. Groups of mice were euthanized on post-infection days 2 and 7 and the bacterial loads in their wound beds and spleens were assessed. Significant differences were not observed (**Figure 1**), indicating that neither overexpression nor absence of Pel and Psl altered the ability of *P. aeruginosa* to establish an infection and survive in this *in vivo* model. While bacteria were detected in the spleens of some mice, they were low in number and not significantly different between groups. This likely represents a low level of transient systemic spread, which did not result in any morbidity or mortality.

**Figure 2:**
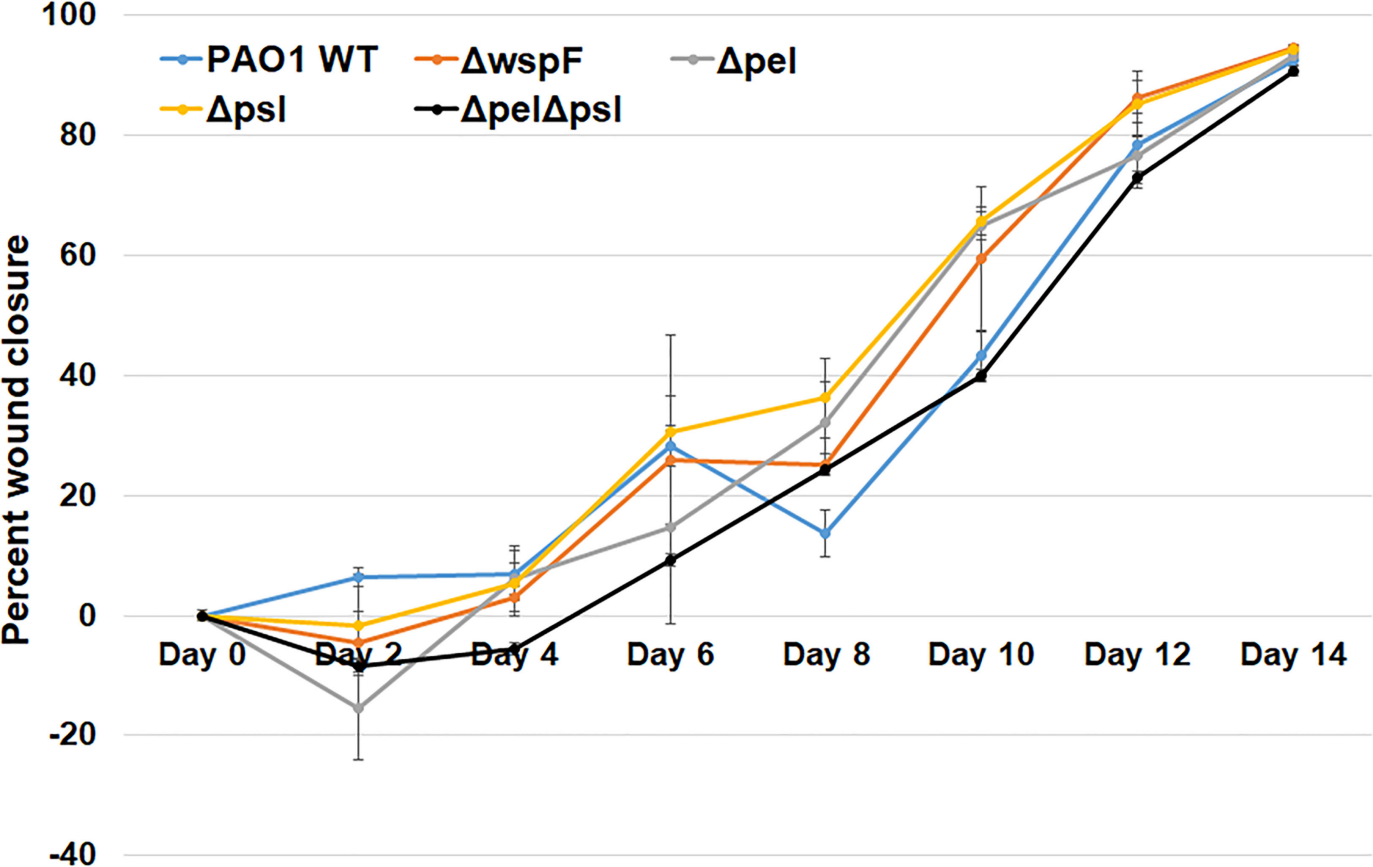
No significant differences in wound closure rates were observed between groups. Mice were wounded and infected with indicated *P. aeruginosa* strains. Every 2 days they were anesthetized and their wounds were imaged and measured. One-way analysis of variance and a Tukey’s multiple comparison test was used to test for differences between groups. n=4-8 mice/strain.

Biofilms are thought to impact healing by acting as mechanical barriers that impede re-epithelialization and by causing a perpetual state of inflammation (Watters et al., 2013; Zhao et al., 2013). To determine whether wound closure was affected by the overexpression or absence of Pel and Psl, we measured the area of infected wounds daily for 14 days. We saw that wound closure rates were similar across all infection groups, indicating that Pel and Psl were not significant contributing factors to wound resolution in this model (**Figure 2**). These observations are somewhat consistent with those of Pestrak et al., who used a similar mouse model to examine the impact of Pel and Psl on *P. aeruginosa* wound infection (Pestrak et al., 2019). While the investigators did see significantly lower bacterial loads in wounds infected with PAO1Δ*pel/*Δ*psl* than with PAO1 WT at 2 and 3 days post-infection, the differences were not significant by 4 days post-infection and wound closure was not assessed (Pestrak et al., 2019).

Overexpression of Pel and Psl by the PAO1Δ*wspF* mutant resulted in biofilms with significantly more biomass *in vitro* (Hickman et al., 2005) (Borlee et al., 2010). However, we observed that infection loads and wound closure rates were similar to those caused by the other strains *in vivo*. In a porcine, full thickness burn wound model, PAO1Δ*wspF* caused a greater bacterial burden in wounds that healed more slowly than those infected with PAO1 (Pestrak et al., 2018). However, these differences were only seen after 35 days post-infection. At early time points (7 days post-infection for bacterial load, and 7 and 14 days post-infection for wound size) there was no statistical difference between PAO1Δ*wspF* and the PAO1 parent strain (Pestrak et al., 2018). Taken together, these current and previous findings suggest that in a more acute murine wound model Pel and Psl have little impact on virulence, but in a more chronic pig model they appear to become important later in infection and their overproduction may impact the ability of wounds to heal.

### Absence of Pel and Psl Alters Spatial Properties of Biofilm Aggregates in Wounds

The sizes and shapes of biofilms grown *in vitro* can vary due to a number of factors, and the production of Pel and Psl has been shown to contribute to this variation in some *in vitro* models. For example, in flow cell experiments, Colvin et al. observed distinct differences in the structures of Pel and Psl mutant biofilms in comparison to WT PAO1 biofilms after 5 days of growth (Colvin et al., 2012). While the *pelA* mutation had virtually no effect on microcolony development, a lack of Psl resulted in an absence of microcolony development and reduced biomass. However, while biofilm structure has been heavily scrutinized in flow cells, it is unclear how or if alteration in biofilm structure relates to infection. As opposed to biofilms formed unperturbed *in vitro*, biofilms in infection are typically present as aggregates that can vary greatly in size (Bjarnsholt et al., 2013; Jennings et al., 2021). To determine if changes to the exopolysaccharides produced would affect the number or size of aggregates in wounds, we performed imaging analysis on tissue from wounds infected with PAO1 and strains lacking Pel and/or Psl.

**Figure 3:**
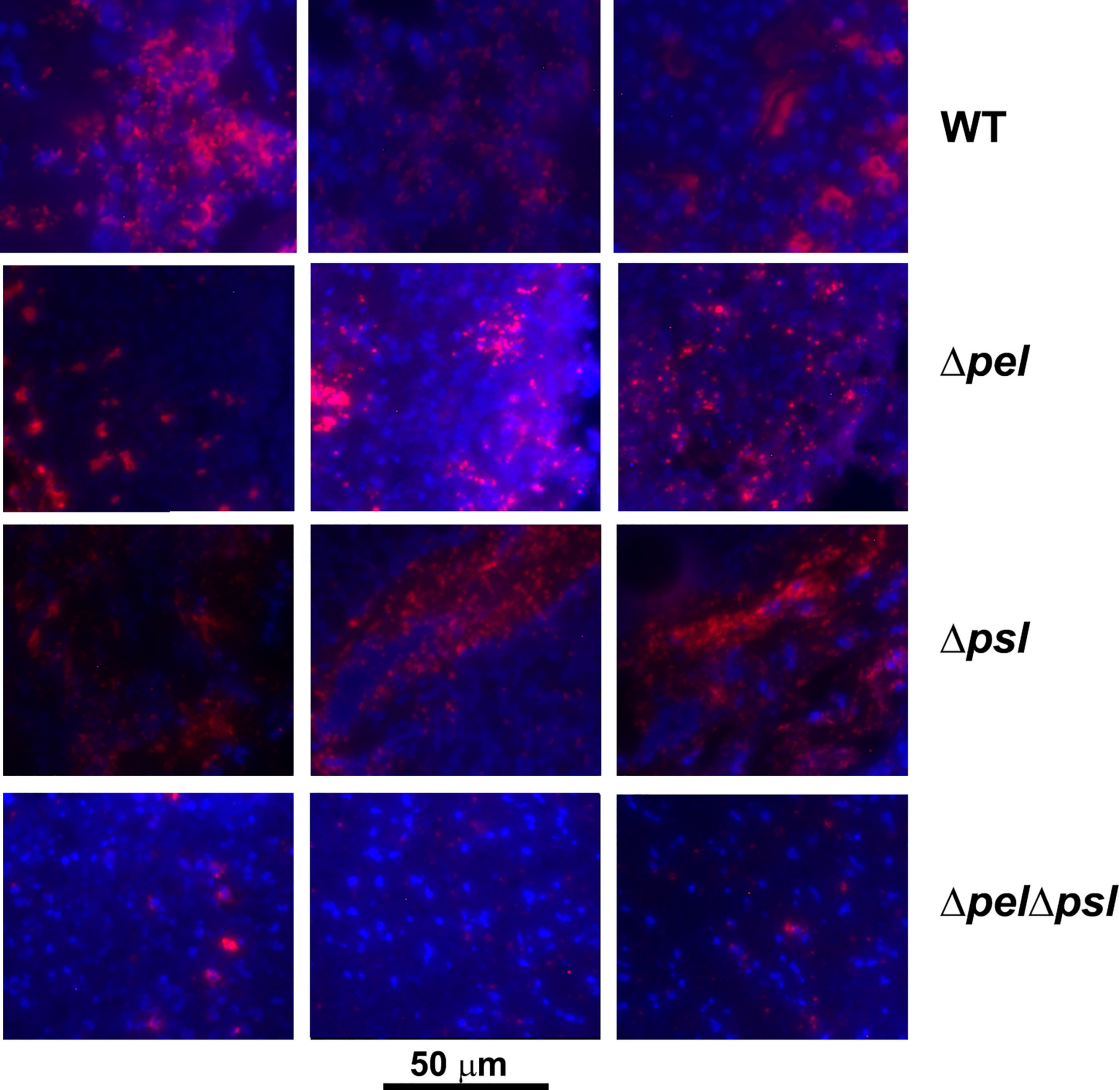
Representative images of murine wound tissue infected with PAO1 exopolysaccharide mutants (red: Alexa Fluor^®^ 594) embedded in host tissue (blue:DAPI). Mice were wounded and infected with indicated *P. aeruginosa* strains. After 10 days, wound tissue was harvested and processed for immunohistochemistry. Three representative images are shown from different mice and different sections to highlight the variability of aggregates observed.

The spatial properties of biofilm aggregates in mouse wounds were examined by performing immunohistochemistry with a *P. aeruginosa* antibody on sections from wounds that had been infected with PAO1 or exopolysaccharide mutants for 10 days (**Figure 3**). We chose this time point based on our experience with this model, were aggregates are difficult to visualize until at least 10-12 days post infection (Dalton et al., 2011; Watters et al., 2013). To ascertain whether the antibody bound the strains similarly, we methanol fixed planktonic cells on slides, and then performed immunohistochemistry as described in the manuscript. We imaged the slides and performed analysis in ImageJ to determine if the antibody signals on cells were similar and if similar numbers of cells were labelled with antibody (**Supplemental Figure 1**). While the average antibody signal appeared to be less for all of the mutant strains in comparison to PAO1, the differences were not significant and did not visually appear different. We also did not see a significant difference in the number of cells (identified by DAPI) that were labelled by the antibody as determined by the mean of Pearson’s coefficient.

**Figure 4:**
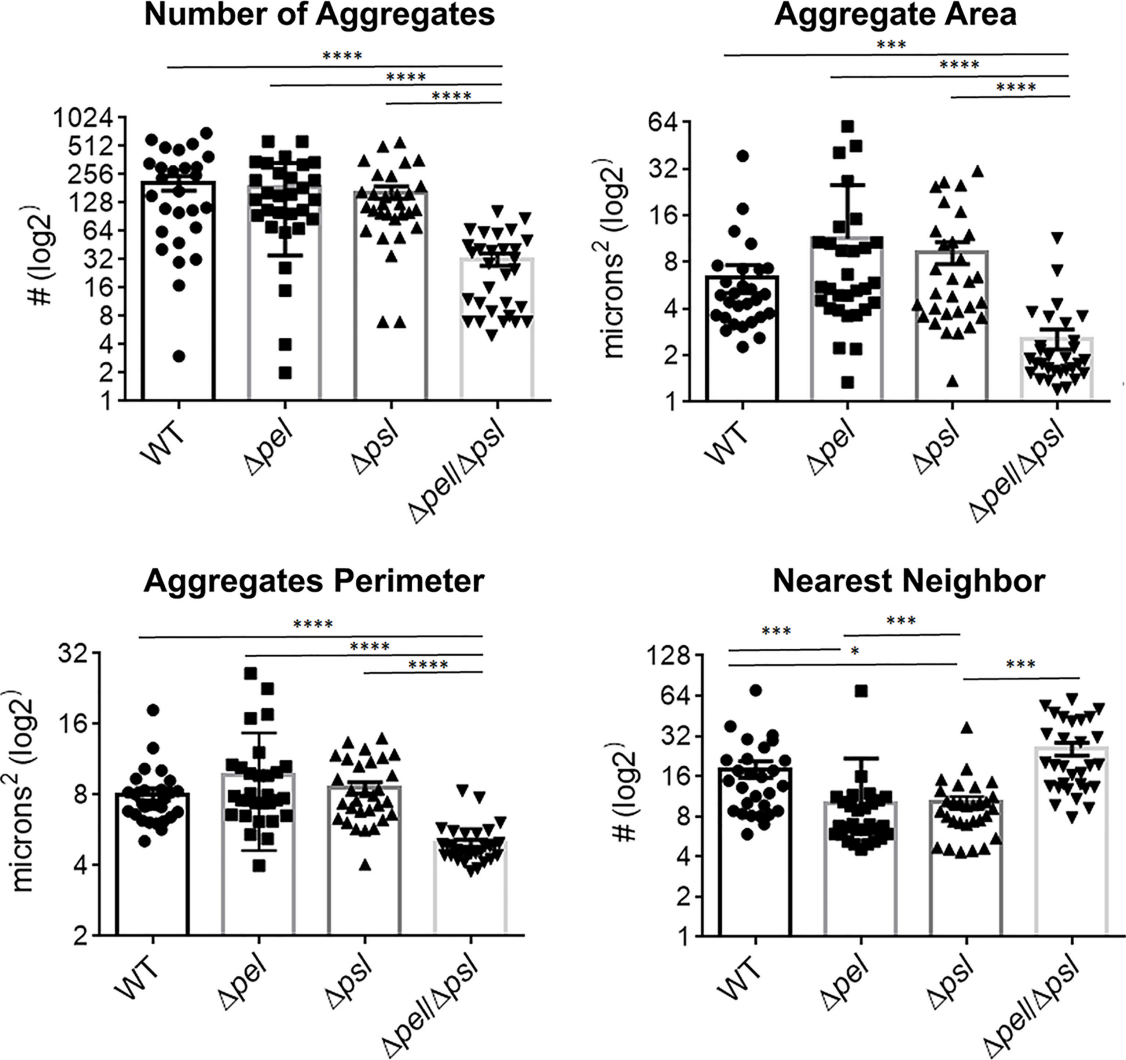
*P. aeruginosa* aggregate characteristics of exopolysaccharide mutants in wound infections. Mice were wounded and infected with indicated *P. aeruginosa* strains. After 10 days, wound tissue was harvested and processed for immunohistochemistry. Images of aggregates in wound tissue were acquired and analyzed using image analysis. One-way analysis of variance and a Dunn’s multiple comparison test was used to test for differences between groups. *, P<0.05; ***, P<0.001; ****, P<0.0001. n=30 images per strain (10 images/mouse and 3 mice/strain).

Using image analysis, we analyzed 30 images from each infection group (i.e. 10 images per mouse and 3 mice infected with each strain) and determined the number, size and spatial distribution of 17,827 bacterial aggregates within the wound tissue (**Figure 4**). We found that the lack of both Pel and Psl resulted in fewer and smaller aggregates. This was surprising considering that CFU analysis on infected tissue showed similar bacterial loads (**Figure 1**) and likely indicates that more of the PAO1 Δ*pel*Δ*psl* are present in the tissue as single cells, which are below the threshold of detection in our image analysis. We also found that aggregates of the double mutant were spaced further apart than were aggregates of either the single mutant strains or the wild-type (**Figure 4**, Nearest Neighbor), which makes sense as they are fewer and smaller. Similarly, as aggregates of the single knockouts were significantly larger than those of the double mutant, we also saw that they were spaced more closely together (**Figure 4**, Nearest Neighbor). Surprisingly, the spacing between aggregates of the double mutant and the wild-type were statistically indistinguishable. This is likely due to these two groups having the largest distances between aggregates, as well as the largest standard deviation between measurements.

**Figure 5:**
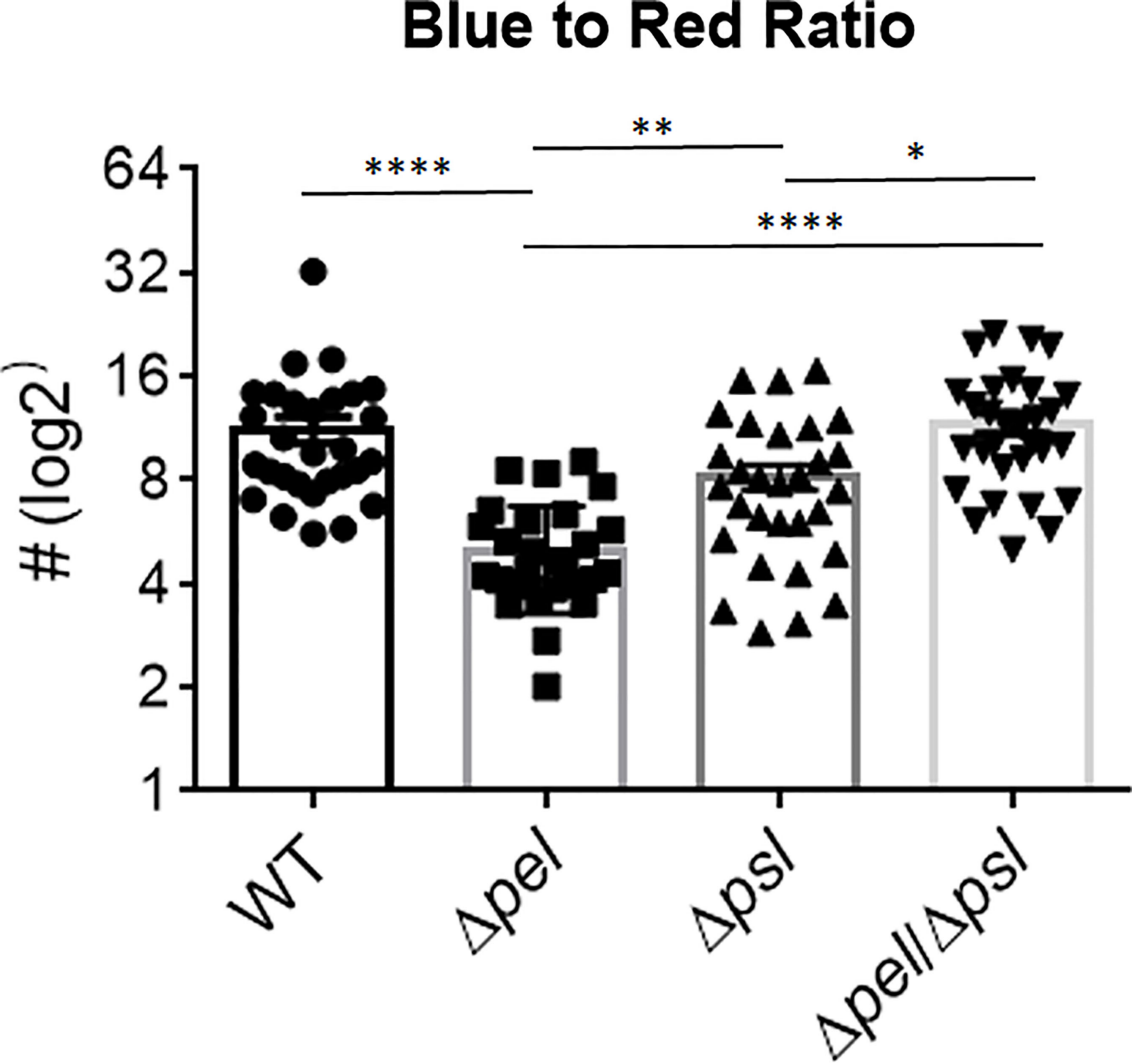
Pel deletion results in a smaller host to bacteria ratio. Mice were wounded and infected with indicated *P. aeruginosa* strains. After 10 days, wound tissue was harvested and processed for immunohistochemistry. DAPI (blue) signal was used as a proxy for ‘host’ and Alexa Fluor^®^ 594 (red) was used as a proxy for bacteria. Images of aggregates in wound tissue were acquired and analyzed using image analysis. One-way analysis of variance and a Dunn’s multiple comparison test was used to test for differences between groups. *, P<0.05; **, P<0.01; ****, P<0.0001. n=30 images per strain (10 images/mouse and 3 mice/strain).

We also investigated whether the absence of Pel or Psl would affect the abundance of host cells near bacterial aggregates. We measured the relative abundance of host material by taking the ratio of blue (DAPI; host cells) to red (*P. aeruginosa*) light intensity (**Figure 5**). Surprisingly, we found that deletion of Pel, but not Psl or even deletion of both exopolysaccharides, resulted in a lower host cell to bacterial cell ratio. If we assume that a large portion of these host cells are immune infiltrate, one explanation for this observation is that the lack of Pel results in a less active immune response. However, the mutant lacking both Pel and Psl did not show a similarly lower host cell to bacterial cell ratio. This could suggest the reduced host-cell signal is due to Psl being employed as the primary matrix scaffold during infection with PAO1Δ*pel*. Psl production by mucoid strains of *P. aeruginosa* has been shown to stimulate inflammation in murine lungs (Jones and Wozniak, 2017), thus it is unclear why Psl production in murine wounds would not also cause inflammation. Additionally, it is possible that these host cells are not immune infiltrate, but other cell types (e.g. epithelial, keratinocyte, fibroblasts) and their abundance may be a reflection of other factors within the infection microenvironment that are different between infection groups. For example, perhaps overproduction of Psl by the Δ*pel* strain is detrimental to host cells, causing cell death in the infected wound tissue. Psl production by *P. aeruginosa* in a keratitis infection model is was shown to be involved in a ‘dead-zone’ around aggregates, which was thought to involve neutrophil extracellular traps (Thanabalasuriar et al., 2019). As DAPI indiscriminately stains both the intracellular and extracellular DNA of both host cells and bacteria, future experiments will be needed to determine if a similar process is occurring in wound infections.

### The Lack of Pel and Psl Reduces Antibiotic Tolerance in Wounds

**Figure 6:**
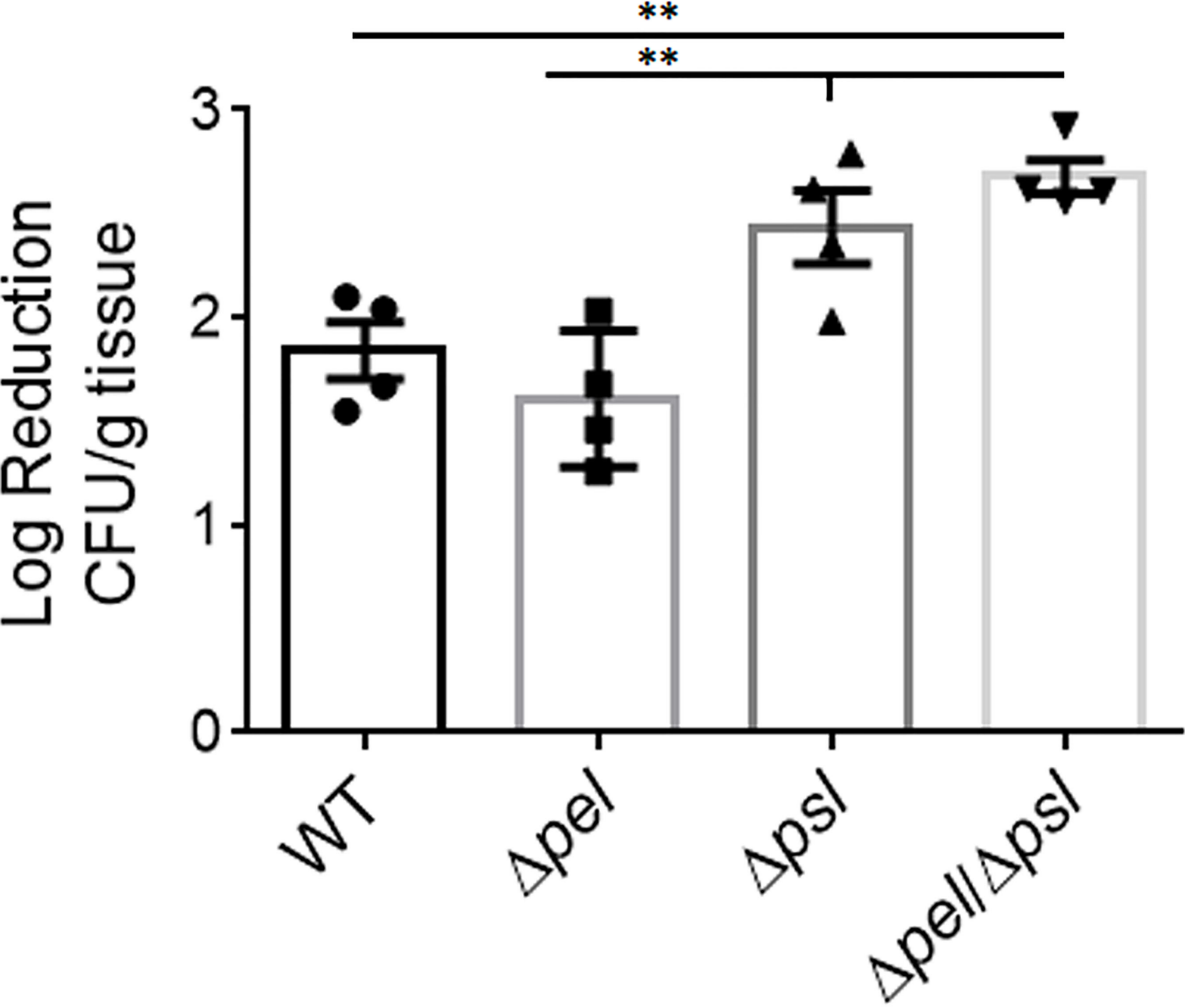
Deletion of both Pel and Psl results in significantly reduced tolerance of *P. aeruginosa* to gentamicin sulfate. Mice were wounded and infected with indicated *P. aeruginosa* strains. After 4 days, wound tissue was harvested and treated *ex vivo* with gentamicin sulfate. One-way analysis of variance and a Dunn’s multiple comparison test was used to test for differences between groups. **, P<0.01; n=4 mice/strain).

The contribution of the biofilm matrix to survival of antibiotic treatment has been well studied *in vitro*. Although there have been confounding reports regarding mechanisms, it is generally accepted that exopolysaccharides produced by *P. aeruginosa* can help confer protection against antibiotics, especially to aminoglycosides, and that tolerance typically increases with greater biomass (Colvin et al., 2012; Tseng et al., 2013; Goltermann and Tolker-Nielsen, 2017; Ray et al., 2017; Jennings et al., 2021). Given the smaller and fewer aggregates created by the Pel and Psl mutant in our *in vivo* model, we sought to determine if antibiotic tolerance was affected (**Figure 6**). We found that wound beds infected with the double mutant, which had the smallest aggregates of all strains studied, were significantly less tolerant to gentamicin sulfate than WT PAO1 and the PAO1Δ*pel* strain, when treated *ex vivo*. We also saw that the Psl mutant was significantly less tolerant to gentamicin sulfate treatment than the PAO1Δ*pel* strain. As there was no difference in aggregate size between these two strains, it is clear that tolerance to gentamicin was not solely due to biomass. It is possible that the physical or chemical make-up of the PAO1Δ*psl* aggregates or the way they associate with host components decreases their tolerance.

## Materials And Methods

### Bacterial *Strains* and Growth Conditions

Bacterial strains used are described in **Table 1**. Frozen bacterial stocks were stored at -80°C prior to culture. Using an inoculating loop, the bacterial stock was streaked onto LB (Luria-Bertani) agar and grown overnight at 37°C. One colony of the resulting streak was inoculated into 10 mL of LB broth and grown for 16 hours at 37°C in a 125 mL Erlenmeyer flask, with shaking at 220 RPM. Following incubation, overnight *P. aeruginosa* cultures were diluted to an optical density of 0.4 at 600 nm (OD_600_). After that, 1 mL of the bacterial culture was prepared by centrifugation (10,000 X G for 5 minutes), washed and re-suspended in 1 X Phosphate Buffered Saline (PBS).

### Murine Chronic Wound Infection Model

Our murine surgical excision wound model has been previously described (Brown and Greenhalgh, 1997; Rumbaugh et al., 2009; Wolcott et al., 2010; Dalton et al., 2011; Fleming et al., 2017; Fleming et al., 2020; Redman et al., 2021). Briefly, mice were anesthetized by intraperitoneal injection of ketamine and xylazine. After a surgical plane of anesthesia was reached, the backs were shaved and administered a full-thickness, dorsal excisional skin wound to the level of panniculus muscle with surgical scissors. Wounds were then covered with a semipermeable polyurethane dressing (OPSITE dressing; Smith & Nephew^®^), under which 10^4^ bacterial cells were injected into the wound bed, and biofilm formation was allowed to proceed for the indicated time, after which the animals were sacrificed and their wound beds harvested for colony forming unit (CFU) analysis or imaging.

### Imaging of Infected Mouse Wound Tissue

Mice were infected with the strains indicated as described above. After 10 days of infection, wound tissue was harvested, fixed in 10% formalin, embedded in paraffin, and cut into 10 µm sections. Sections were de-paraffinized, and labelled with an anti-*Pseudomonas* primary antibody (Chicken anti-*P. aeruginosa*; Abcam, PLC: ab74980) coupled to a red-fluorescent 2°antibody (Goat anti-chicken IgY H&L; Abcam, PLC: ab150176), and mounted with DAPI (ProLong^®^ Diamond Antifade ThermoFisher: P36962) for immediate imaging with a Nikon eclipse TS 100-F Epifluorescence microscope.

### Wound Bacterial Load Quantification

After 2 or 7 days of infection, entire wound beds were harvested, weighed, and placed into FischerScientific™ 2mL Pre-Filled Bead Mill Tubes with 1 mL of PBS and homogenized at 5 m/s for 60 seconds using a FastPrep-24™ MP Biomedical Benchtop Homogenizer. The resulting homogenates were then serially diluted 1:10 in PBS and plated on Pseudomonas Isolation Agar (Difco™). Plates were incubated at 37°C overnight, after which the bacterial loads were determined by CFU quantification.

### Wound Closure Rate Quantification

Wounds were measured by daily imaging with a SilhouetteStar™ (ARANZ Medical) wound imaging camera. Percent wound closure was determined by subtracting the area of the wound measured on each day from the area on day 0, dividing by the area on day 0, and multiplying by 100.

### *Ex Vivo* Antibiotic Tolerance

Equal-sized tissue sections from the wounds of infected mice were suspended in 200 µg/mL gentamicin sulfate (Sigma Aldrich) or PBS for 5 hours. Antibiotic treatment was removed with 3 washes of PBS. The samples were then homogenized as described in wound bacterial load quantification, serially diluted and plated on *Pseudomonas* isolation agar to quantitate CFU/g. The number of cells viable after antibiotic treatment was compared to the number of cells viable after the PBS treatment to determine a log reduction.

### Image Analysis of Infected Wound Tissue

The image analysis for this project was done in Fiji (Schindelin et al., 2012) and Python. In Fiji, images of infected mouse wound sections were split into red and blue channels to create 2 greyscale 8-bit images. The red and blue channels correspond to the Alexa Fluor^®^ 594 secondary antibody used to detect bacteria, and the DAPI DNA binding dye, respectively. From each of the images, Intensity values and shape parameters were obtained using Fiji’s “Measure” function and used as a measure of total bacteria and total host wound material. Then, a threshold was applied to the red (“bacteria”) channel to create a black and white 8-bit image. From the thresholded image, area and center of mass position were obtained for all bacterial clusters using Fiji’s built-in “Analyze Particle…” function.

Nearest neighbor distance is defined as the two-dimensional Euclidean distance from the bacterial cluster’s center-of-mass position to the closest neighboring bacterial cluster’s center-of-mass position. The following Python function returns a list of the nearest neighbors for all the aggregates given a 2D list of [“x”,”y”] coordinates.


$$\begin{array}{c} def\;NN\left(dlist\right)\\ NN\_list=\left[\right]\\ for\;i\;in\;range\left(len\left(dlist\right)\right):\\ NN=\;100000000\\ for\;j\;in\;range\left(len\left(dlist\right)\right):\\ if\;i!=j:\\ temp\;=\;\left(\left(\left(dlist\left[i\right]\left(0\right)-dlist\left[j\right]\left(0\right)\right)**2\right)+\left(dlist\left[i\right]\left(1\right)-dlist\left[j\right]\;\left(1\right)\right)**2\right)**.5\\ if\;temp\;<\;NN:\\ NN=temp\\ NN\_list.append\;\left(NN\right)\\ return\;NN\_list \end{array}$$

### Statistical *Analysis*

GraphPad Prism (GraphPad Software, Inc.) was used for statistical analysis. Specific statistical tests used are provided in the figure legends.

### Vertebrate Animal Use

All animal experiments were carried out in strict accordance with the recommendations in the Guide for the Care and Use of Laboratory Animals of the National Institutes of Health. The protocol was approved by the Institutional Animal Care and Use Committee of Texas Tech University Health Sciences Center (Protocol Number: 07044).

### Immunohistochemistry of Planktonic Cells

Planktonic cultures were grown overnight in Luria Bertani Broth at 37C, with shaking at 200 rpm. Overnight cultures were diluted to 10^7^ cfu/mL and placed dropwise on a slide. Samples were allowed to dry and subsequently methanol fixed. Prepared slides were incubated overnight at 4C in Primary Antibody (Abcam ab74980 Chicken Anti-Pseudomonas aeruginosa) diluted in PBS with 4% Dried Milk. Slides were washed with PBST before being incubated for 1 hour at room temperature in Secondary antibody (Abcam ab105176 Goat Anti-Chicken with Alexa594) diluted in PBST. Slides were washed following secondary incubation and prepared for imaging with ProLong Diamond Anti-Fade Mountant with DAPI (Invitrogen). Images were captured using a Nikon Eclipse 80i Fluorescent Scope with DS-Fi1 camera. Analysis was performed using ImageJ. For automated counting of antibody-positive cells, thresholds were applied to the images using the IJ_IsoData threshold function, followed by Analyzing Particles. For Corrected Total Fluorescence per area, integrated density was summed for each image, mean background subtracted, and the resulting difference was divided by the total fluorescence area. For colocalization, the Colocalization Threshold function was applied to determine P and Pearson’s coefficient for each antibody-DAPI image pair.

## Discussion

*P. aeruginosa* is an opportunistic pathogen that uses at least three different exopolysaccharides, Pel, Psl and alginate, in its biofilm EPS. The production of these exopolysaccharides depends on the specific bacterial strain and on growth conditions (Colvin et al., 2012). However, it is thought that the primary exopolysaccharides produced in wounds are Pel and Psl (Wozniak et al., 2003). While the roles of Pel and Psl have been characterized to a large degree *in vitro*, how this characterization correlates with their roles *in vivo* is poorly understood. From *in vitro* studies it is apparent that Pel and Psl serve many functions including surface attachment, structural integrity of the biofilm, and antimicrobial and immune tolerance (Maunders and Welch, 2017), but few studies have sought to determine if these functions extend to infection.

Here we used a mouse wound model to characterize infections produced by a WT strain of *P. aeruginosa*, compared to PAO1 mutant strains that either did not produce Pel and/or Psl, or over-produced them (**Table 1**). We compared overall infection progression in mouse wounds, assessing bacterial load and wound closure as surrogate markers for virulence at 2 and 7 days post-infection. We discovered no significant differences between the bacterial loads in the wounds or spleens of mice infected with any of the bacterial strains studied. It should be noted however, that despite similar wound bioburden, infection with the mutant lacking both Pel and Psl resulted in no detectable bacteria in the spleens at either time point. This is the only strain in which this was the case, which could indicate that lacking both Pel and Psl leads to decreased hematogenous spread (i.e. decreased dispersal potential, decreased protection against the immune system, etc.). However it is more likely that the low number of bacteria detected in the spleens represents transient spread. We also saw no difference in wound closure rates over 14 days between infection groups.

Given the reported importance of Pel and Psl *in vitro*, it was unexpected that our data showed that their absence did not grossly affect the progression and resolution of *P. aeruginosa* wound infection. Our *in vivo* results are somewhat consistent with those reported by Pestrak et al. (2019). Using a similar mouse excisional wound model, they compared bioburden in wounds infected with either PAO1 or PAO1Δ*pel* Δ*psl* at 1, 24, 48, 72, or 96 hours post-infection. They observed significant reductions in the bacterial load of wounds infected with PAO1Δ*pel* Δ*psl* at 48 and 72 hours post-infection, but by 96 hours, the difference was not significant. Wound size was not measured, so it is unclear if there were differences. They concluded that loss of Pel and Psl impacted initial colonization, but did not affect the long-term outcome of an infection (Pestrak et al., 2019). However, data from another study by Pestrak et al. showed that infection of porcine wounds with the Pel/Psl overproducing strain Δ*wspF* caused a different infection outcome than with WT PAO1 (Pestrak et al., 2018). In the porcine wound infection model, significant differences in the bacterial load of wounds infected with PAO1 versus Δ*wspF* were detected at 7, 14 and 35 days post-infection. However, these differences were not consistent. At 7 and 14 days post-infection, wounds infected with WT PAO1 had higher bioburden, while at 35 days the PAO1Δ*wspF* infected wounds had higher bioburden. Importantly, wounds infected with PAO1Δ*wspF* were significantly larger at 35 days post-infection than those infected with WT PAO1 (Pestrak et al., 2018). This led the authors to suggest that PAO1Δ*wspF* caused a more persistent and severe infection in pigs, impairing wound healing. We did not see this with PAO1Δ*wspF*, which could be primarily due to the animal model used. Porcine wound models are superior to mouse models because the dermal structure and mechanisms for healing much more closely resemble that of humans (Zindle et al., 2021). Therefore they are thought to more accurately model chronic infection. Taken together, these results demonstrate that the importance of Pel and Psl *in vivo* varies considerably between models and may only be apparent after a long period of chronic infection.

Since exopolysaccharides affect biofilm structure *in vitro*, we sought to investigate whether the spatial structure of the multicellular bacterial aggregates would be influenced by the loss of Pel and/or Psl *in vivo*. Using immunofluorescence microscopy and image analysis, we determined that infections with the mutant lacking both Pel and Psl exhibited fewer, smaller aggregates with greater spacing between aggregates. The fact that these differences in aggregate spatial structure exist despite similar bacterial loads suggests that much of the population of the Pel/Psl deficient cells exist as unaggregated single cells that are difficult to visualize with standard imaging techniques in wound tissue (Fleming et al., 2020). If true, one would expect these single cells to be more susceptible to clearance by the immune system. However, the similar bacterial loads indicate that this was not the case. We also saw that the intensity ratio of the blue (DAPI; host cells) and red (*P. aeruginosa*) signal in the immunofluorescent images was not affected by the loss of both Pel and Psl, which would be expected if there was a large difference in immune infiltrate. Instead, we saw that deletion of Pel alone resulted in a lower host cell to bacterial cell ratio. At this time we cannot explain this finding. However, it will be important in future studies to clearly identify the types of host cells that are less abundant around PAO1Δ*pel* aggregates.

Finally, we sought to investigate what role Pel and Psl play in antibiotic tolerance in wounds. Exopolysaccharides are clearly involved in the increased tolerance biofilm cells display to antibiotics. Specifically, the polycationic polymer Pel has especially been linked to tolerance to positively charged aminoglycoside antibiotics (Colvin et al., 2011; Jennings et al., 2015b; Jennings et al., 2021). Psl has also been shown to play a protective role against some antibiotics, but the mechanism is less understood (Billings et al., 2013). Thus we hypothesized that aggregates devoid of at least Pel would be more susceptible to killing by aminoglycosides. Our data supported this hypothesis as we saw that PAO1Δ*pel*Δ*psl* in wound tissue displayed a reduced ability to survive gentamicin treatment; however, we also saw that PAO1Δ*pel* did not exhibit this same tolerance. Instead, PAO1Δ*psl* in wound tissue was similarly tolerant to gentamicin treatment as PAO1Δ*pel*Δ*psl*. This result is perplexing since Pel is thought to play the major role in protection against aminoglycoside antibiotics. It also suggests that Psl may play a role in protecting *P. aeruginosa* from antibiotics *in vivo*, which has also been observed by other investigators *in vitro* (Billings et al., 2013). Other factors such as metabolism of the biofilm population may also be involved in our observations. Future studies will aim to better understand the interactions between the exopolysaccharides produced and efficacy of different classes of antibiotics.

There were several limitations to this study. Firstly, although there is evidence to support the production of Pel and Psl in aggregates during infection (Ray et al., 2017; Jennings et al., 2021), extensive characterization of *in vivo* exopolysaccharide production has not been reported, and we did not specifically verify that Pel and Psl are produced in mouse wounds. This is because the methods and reagents to specifically identify these exopolysaccharides amongst the myriad of host components present *in vivo* are still not readily available. We also acknowledge that since PelA is a deacetylase, it is possible that PAO1Δ*pel* could produce an acetylated version of Pel, which isn’t recognized by Pel antisera. To explore this, future experiments should include other *pel* mutants. Future studies will also benefit from thorough assessment of which exopolysaccharides are produced during different stages of wound infection. Additionally, while Pel and Psl are thought to be the major exopolysaccharides produced by *P. aeruginosa* in chronic wound infections (Wozniak et al., 2003), alginate is also produced by PAO1 and its involvement cannot be discounted. It is possible that alginate production by one or more of these mutants can help explain some of our findings. Another limitation is that the imaging studies were performed at one time point, yet the EPS composition of *P. aeruginosa* may change over time *in vivo*, as it does *in vitro* (Yang et al., 2011). For example, one exopolysaccharide may dominate the EPS at early stages of the infection, and other exopolysaccharides may dominate at later stages of infection. If this is the case, the results presented herein should be taken as indicative of the contributions of polysaccharides to established wound infections. Going forward, a time course examination of aggregate spatial properties during the course of infection may help resolve some of these uncertainties.

To conclude, in this study we attempted to define the roles of the Pel and Psl polysaccharides in chronic wound infection with *P. aeruginosa*, particularly in respect to bacterial load, wound closure rates, aggregate size and spacing, and antibiotic tolerance. We found that, despite similar bacterial loads and wound closure rates, the bacterial aggregates resulting from infection with a *P. aeruginosa* strain that is incapable of producing both Pel and Psl were smaller and fewer, and the cells were more susceptible to gentamicin sulfate. We also showed that deletion of Pel alone likely resulted in lesser immune cell recruitment, despite the double mutant showing similar host cell recruitment as the wild type strain. While this study takes a step forward in investigating *in vivo* polysaccharide production in chronic *P. aeruginosa* wound infection, further work is needed to determine how these affects change temporally over the course of an infection, the potential impact of alginate production, and the identity of the host cells involved.

## Data Availability Statement

The raw data supporting the conclusions of this article will be made available by the authors, without undue reservation.

## Ethics Statement

The animal study was reviewed and approved by TTUHSC IACUC.

## Author Contributions

DF, KR, and VG contributed to conception and design of the study. BN performed the image analysis. KR performed the statistical analysis. DF wrote the first draft of the manuscript. DF, EV, WR, and BN collected and analyzed data. All authors wrote sections of the manuscript and contributed to manuscript revision, read, and approved the submitted version.

## Funding

This work was supported by grants from the National Institutes of Health (1R01AI121500-01A1, National Institute of Allergy and Infectious Diseases), the National Science Foundation (727544; Biomechanics and Mechanobiology; Civil, Mechanical, and Manufacturing Innovation), to VG; National Institutes of Health (R21 AI137462-01A1), the Ted Nash Long Life Foundation, and The Jasper L. and Jack Denton Wilson Foundation, to KR.

## Conflict of Interest

The authors declare that the research was conducted in the absence of any commercial or financial relationships that could be construed as a potential conflict of interest.

## Publisher’s Note

All claims expressed in this article are solely those of the authors and do not necessarily represent those of their affiliated organizations, or those of the publisher, the editors and the reviewers. Any product that may be evaluated in this article, or claim that may be made by its manufacturer, is not guaranteed or endorsed by the publisher.

## Supplementary Material

Supplementary Figure 1 Comparison of antibody binding to methanol-fixed planktonic cells. **(A)** Examples of immunohistochemistry images from WT PAO1 and PAO1Δ*pel*Δ*psl* methanol-fixed planktonic cells with Alexa594 conjugated *P. aeruginosa* antibody in the first colomn, DAPI staining in the second, and an overlay in the third. **(B)** To alayze the average antibody signal per cell, ImageJ analysis of the mean corrected total fluorescence was divided by the total fluorescent area in fluorescence units/0.24 um^2^, N=3 images per strain. Bars are standard error of the mean and groups were not significant by One-way ANOVA, p < 0.05. **(C)** Mean of Pearson’s coefficient showing that the number of DAPI-stained cells, which were also antibody positive, was not significantly different between strains by One-way ANOVA, p < 0.05. n=3 per strain. P for colocalization is 1.00 for all images.
