# Supplementary figures and images for: Contribution of Pseudomonas aeruginosa Exopolysaccharides Pel and Psl to Wound Infections
Source: Front Cell Infect Microbiol. 2022 Apr 7;12:835754. doi: 10.3389/fcimb.2022.835754 (PMC9021892; doi:10.3389/fcimb.2022.835754)

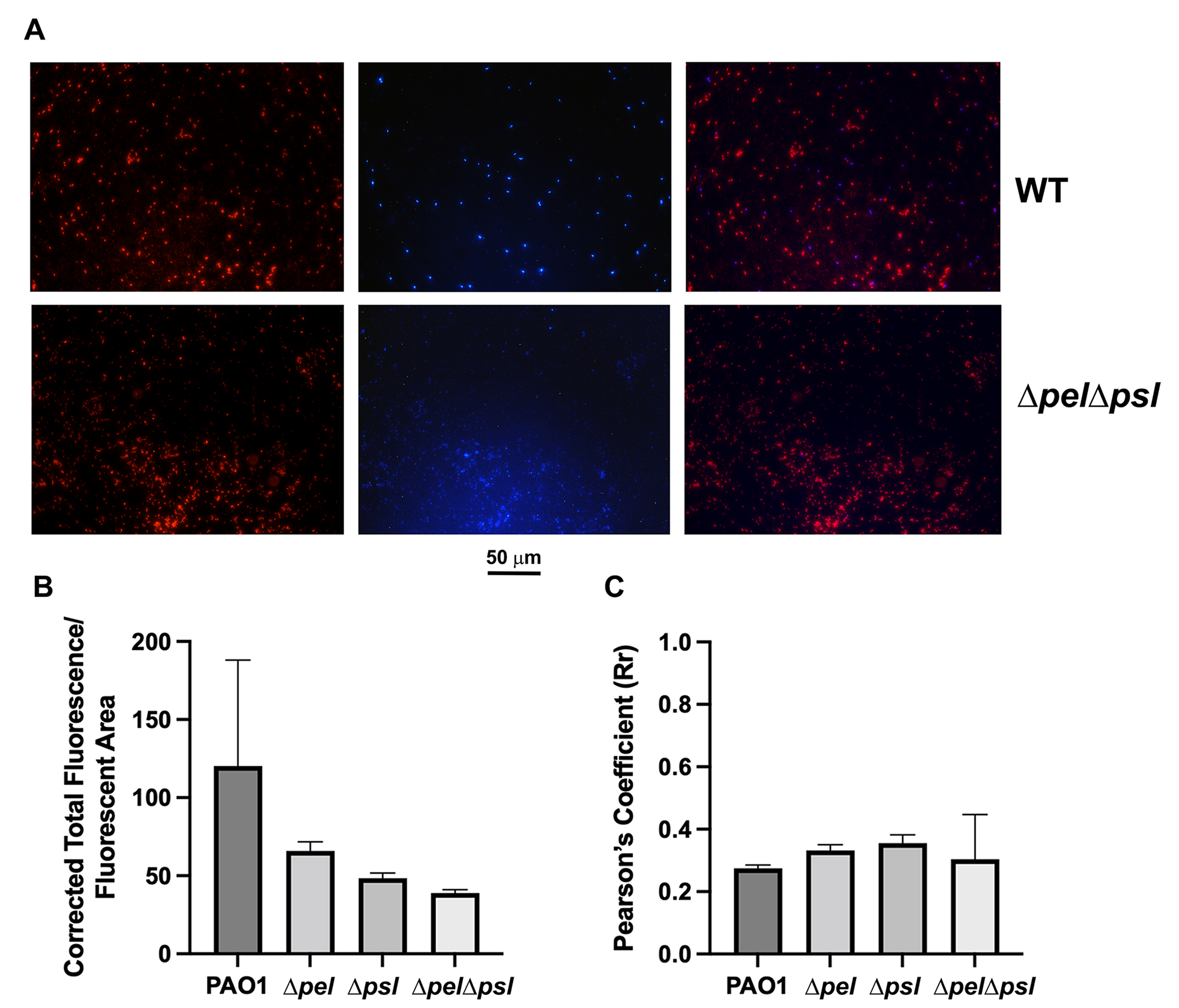

Supplement: Supplementary Figure 1 — Comparison of antibody binding to methanol-fixed planktonic cells. (A) Examples of immunohistochemistry images from WT PAO1 and PAO1ΔpelΔpsl methanol-fixed planktonic cells with Alexa594 conjugated P. aeruginosa antibody in the first colomn, DAPI staining in the second, and an overlay in the third. (B) To alayze the average antibody signal per cell, ImageJ analysis of the mean corrected total fluorescence was divided by the total fluorescent area in fluorescence units/0.24 um2, N=3 images per strain. Bars are standard error of the mean and groups were not significant by One-way ANOVA, p < 0.05. (C) Mean of Pearson’s coefficient showing that the number of DAPI-stained cells, which were also antibody positive, was not significantly different between strains by One-way ANOVA, p < 0.05. n=3 per strain. P for colocalization is 1.00 for all images. [file Image_1.tif]
